# Supplementary material for: “Attractive” Treatment for Abdominal Aortic Aneurysm Repair: Magnetic Localization of Silk-Iron Packaged Extracellular Vesicles
Source: J Funct Biomater. 2025 Oct 22;16(11):395. doi: 10.3390/jfb16110395 (PMC12653497; doi:10.3390/jfb16110395)
Supplement: Supplementary file 1 [file jfb-16-00395-s001.zip › jfb-3880037-supplementary.pdf]

Methods

Extracellular Vesicle Preparation

Two different methods of vesicle isolation were performed, both UC and TFF. After 48 hours of culture, conditioned media (CM) from MSCs was removed and placed in 50 mL conical tubes. For UC, CM was centrifuged at 250 × g followed by 2,500 × g to remove dead cells and cell debris. CM was then filtered through 0.22 μm filters (Millipore Sigma, SLGPM33RS) to remove apoptotic bodies. Following filtering, 34 mL of CM was added to each ultracentrifuge tube (Beckman Coulter, Brea, CA, USA, 326823) and placed on a swinging bucket rotor (Beckman Coulter, SW-28, 117.1 g cases). The CM was subjected to UC at 100,000 × g for 70 minutes at 4°C (Beckman Coulter, L8-70M). Supernatant was removed, and excess protein droplets were aspirated. EV isolate was then resuspended in 600 μL of cold PBS as described previously [15, 16]. For TFF, CM was then added to the feed end of the TFF system (Repligen, Waltham, MA). TFF was used to isolate EVs by flowing media at 100 mL/min through a 300 kDa hollow fiber membrane filter. EVs were obtained in the retentate. Additionally, Dulbecco's phosphate buffered saline (DPBS) (75 mL, Gibco) was used for a buffer exchange to remove residual protein. Isolated EVs were mixed with a concentrated solution of KH_2PO_4 and K_2HPO_4 to create a final solution of 2M potassium phosphate buffer and 1E9 particles/mL concentration EVs, pH = 8.0 for fabricating the SIPEs.

Nanoparticle Tracking Analysis

NTA was performed with a Nanosight (Malvern Panalytical, Malvern, UK, NS300, NTA 3.4, Build 3.4.4) to determine particle counts, i.e., EV counts. For EV isolation counts, an aliquot of 40 μL was mixed with 360 μL of Nanosight water (ultrapure water filtered through 0.22 μm filters) to create a 1:10 dilution. For microparticle releasates, sample was filtered through a 0.22 μm filter to remove large silk debris; releasate samples were also not diluted. For EVs purchased from RoosterBio Inc, an aliquot of 10 μL was mixed with 390 μL of Nanosight water to create a 1:40 dilution. Sample was added to Luerslip syringe to see particles on the Nanosight. Syringe speed was 50 μL/min to read particle counts. Three 45 second particle recordings were taken and averaged to calculate particle counts.

Micro Bicinchoninic Assay (MicroBCA) for Protein Measurement

EVs were diluted to 1E9, 2E9, and 3E9 particles/mL in DPBS. To evaluate concentration of encapsulated protein, RIPA solution (ThermoFisher Scientific) was used and diluted 1:10 (5 mL into 45 mL DPBS). EVs were further diluted into either DPBS or diluted RIPA solution (250 μL of EVs into 250 μL of diluent) . To lyse the membranes, EVs were incubated at 4°C for 30 minutes and vortexed for 30 seconds. A standard curve was made according to the manufacturer's protocols. EVs in DPBS, EVs in diluted RIPA solution, or standards were added to a 96 well plate (150 μL per well) in triplicate and mixed with 150 μL of working reagent. The plate was incubated for 2 hours at 37°C and then read at 562 nm.

Western Blot to Determine EV Surface Markers

EVs at 1E9 particles/mL (15 μL) were boiled in 5 μL of a DTT (ThermoFisher Scientific, 20 μL, 1M, R0861) and Lamelli buffer (60 μL, Bio-rad, Hercules, CA, USA) solution for 5 minutes at 98°C and added to the wells of a 7.5% acrylamide gel. Parent MSC lysate was also used as a control. Gel was run at 25 mA for 75 minutes. The gel was then transferred to nitrocellulose paper for eventual Western Blot analysis. The gel was transferred using two transfer buffer packets (BupH Tris-Glycine mixture, ThermoFisher Scientific, 28380), 200 mL methanol, and 800 mL of ultrapure water) and using a transfer apparatus. Transfer occurred with 70V for 60 minutes. Then the membrane was blocked for 1 hour in 5% milk, 0.1% Tween-20 phosphate buffered saline solution. Blocking solution was diluted 1:2 with PBS. Apolipoprotein B (ApoB) antibody (ThermoFisher Scientific, MA5-15851) was mixed 1:500 in 2.5% milk, 0.05% Tween-20 PBS (10 mL) and added to the membrane. The membrane was then incubated overnight at 4°C.

The next day, the primary antibody was removed, and the membrane was washed with 2.5% milk, 0.05% Tween-20 blocking solution 3 times, 5 minutes each. Then a secondary antibody solution (HRP) was mixed 1:5000 into the blocking solution and added to the membrane for 1 hour at room temperature with constant mixing. The membrane was washed 3 more times, 5 minutes with the first two washes with the milk solution and the final wash with PBS. Then ECL reagents were made. ECL 1 consisted of luminol (200 μL, 500 mM, Millipore Sigma, 120372), p-coumaric acid (180 μL, 90 mM, Millipore, Sigma, C9008), Tris-HCl buffer (5 mL, 1M, pH = 8.3, Lonza, AccuGENE, Rockland, ME, USA, 51237), and ultrapure water (45 mL), and ECL 2 consisted of hydrogen peroxide (32 μL, 30%), Tris-HCl buffer (5 mL, 1M, pH = 8.3), and ultrapure water (45 mL). Blot was then read using ChemiDoc XRS (Bio-Rad).

ExoCheck to Determine EV Surface Markers

For ExoCheck analysis, 50 μg of EVs were used with a ExoCheck Kit (System Biosciences, Palo Alto, CA, USA, EXORAY200B-4). EVs were processed according to manufacturer's protocols. 10X Lysis Buffer was mixed with the sample and diluted to achieve a final concentration of 1X; the sample was vortexed for 15-30 seconds. Then 1 μL of Labeling Reagent was added, and the sample was vortexed again. Sample was mixed for 30 minutes at room temperature. The provided columns for Labeling Reagent removal were prepped by centrifuging at 800 x g for 1 minute, discarding the supernatant, and then adding 400 μL of Column Buffer. This process was performed a total of 5 times. Following preparation, 180 μL of the sample was added to the middle of the packed bed and centrifuged for 2 minutes at 800 x g. After centrifugation, the labelled sample was mixed with 5 mL of Blocking Buffer and mixed by inverting the tube 3 times.

The provided membrane was then added to a clean weigh boat and wetted using ~5 mL of ultrapure water for 2 minutes, ensuring that the membrane was face up by checking for the notch in the upper right corner. The water was then removed and the sample in Blocking Buffer was added to the membrane. The membrane and sample were then mixed overnight at 4°C.

The following day, the lysate and blocking mixture was removed from the membrane. Then 5 mL of the 1X Wash Buffer (which was diluted from the 20X stock in ultrapure water) was added; the membrane was then washed for 5 minutes at room temperature twice. Detection Buffer was prepared by mixing 5 mL of Detection Reagent A and 1.5 μL of Detection Reagent B. Following washing, the Detection Buffer was added to the membrane and mixed for 30 minutes at room temperature. Then the membrane was washed 3 times with Wash Buffer. After washing, the Developer mixture (Advansta Inc., San Jose, USA, CA K12043-C20) was prepared by mixing 1.25 mL of Part A and 1.25 mL of Part B (based off 0.1 mL/cm^2 for the ~21 cm^2 membrane) and added to the membrane. The membrane was then read on ChemiDoc XRS (Bio-Rad).

2.8 EV Uptake Staining

A previous protocol was followed to fix and stain EV uptake samples [28]Media was removed and a stripping buffer (500 μM NaCl and 0.5% glacial acetic acid) was added for 45 s to remove free floating EVs. Cells were washed three times with PBS and then fixed with 3.33% paraformaldehyde (ThermoFisher Scientific) for 20 minutes. Fixation was quenched with 1% (w/v) BSA in PBS solution for 5 minutes, and then cells were washed three times with PBS. Permeabilizing solution (0.1% Triton-X 100 in PBS) was added for 1 minute. Cells were then stained with blocking buffer (300 μL, 1% BSA and 0.1% Triton-X in PBS) containing Phalloidin-488 (1:400, ThermoFisher Scientific, A12379) and Hoechst 33258, pentahydrate (bis-benzimide) (1:1000, ThermoFisher Scientific, H1398) for 1 hour at room temperature. Cells were washed three times with PBS and then mounted on slides for imaging.

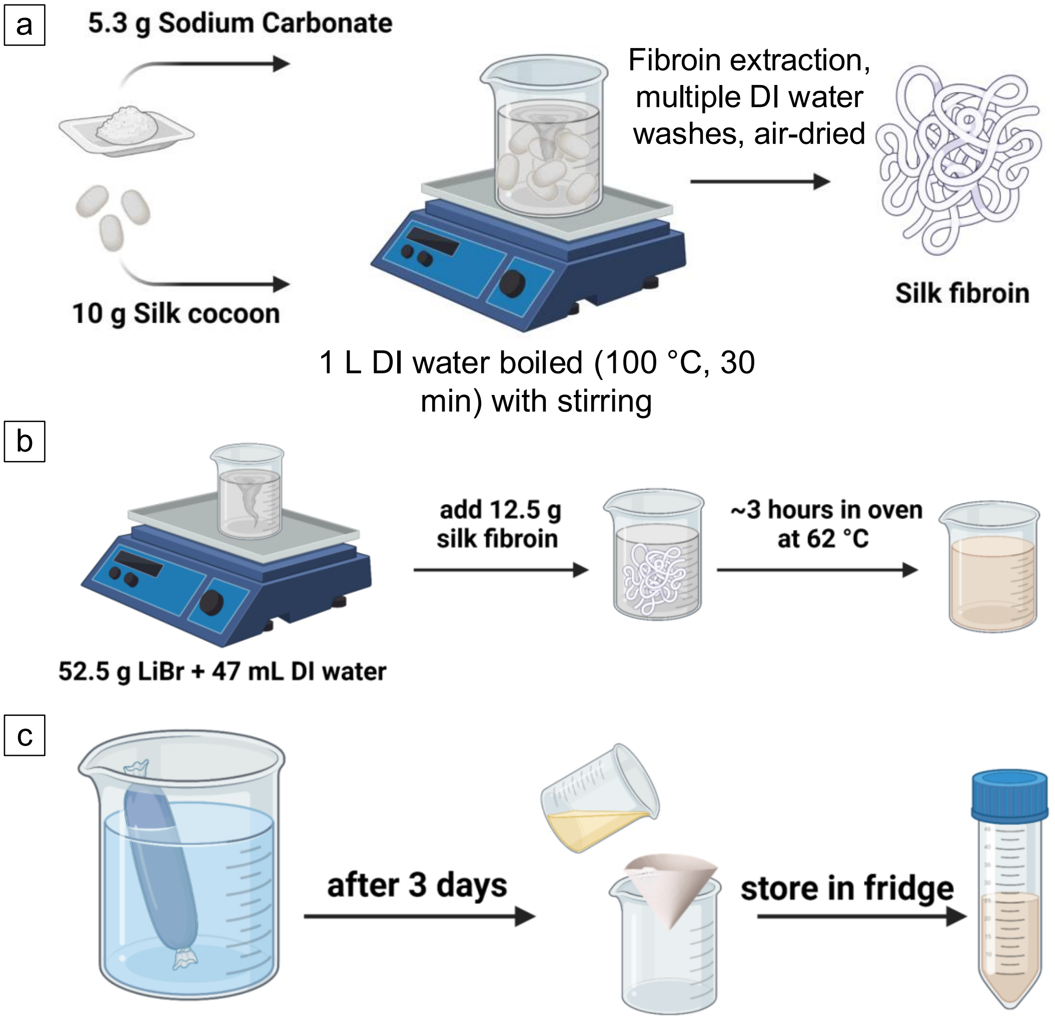

Figure S1. Schematic representation of the synthesis process for RSF solution. The process includes (a) degumming of silk cocoons to obtain fibroin followed by (b) dissolution in a lithium bromide solution. (c) The fibroin solution undergoes dialysis and filtration for purification. Adapted from methods described in [35].

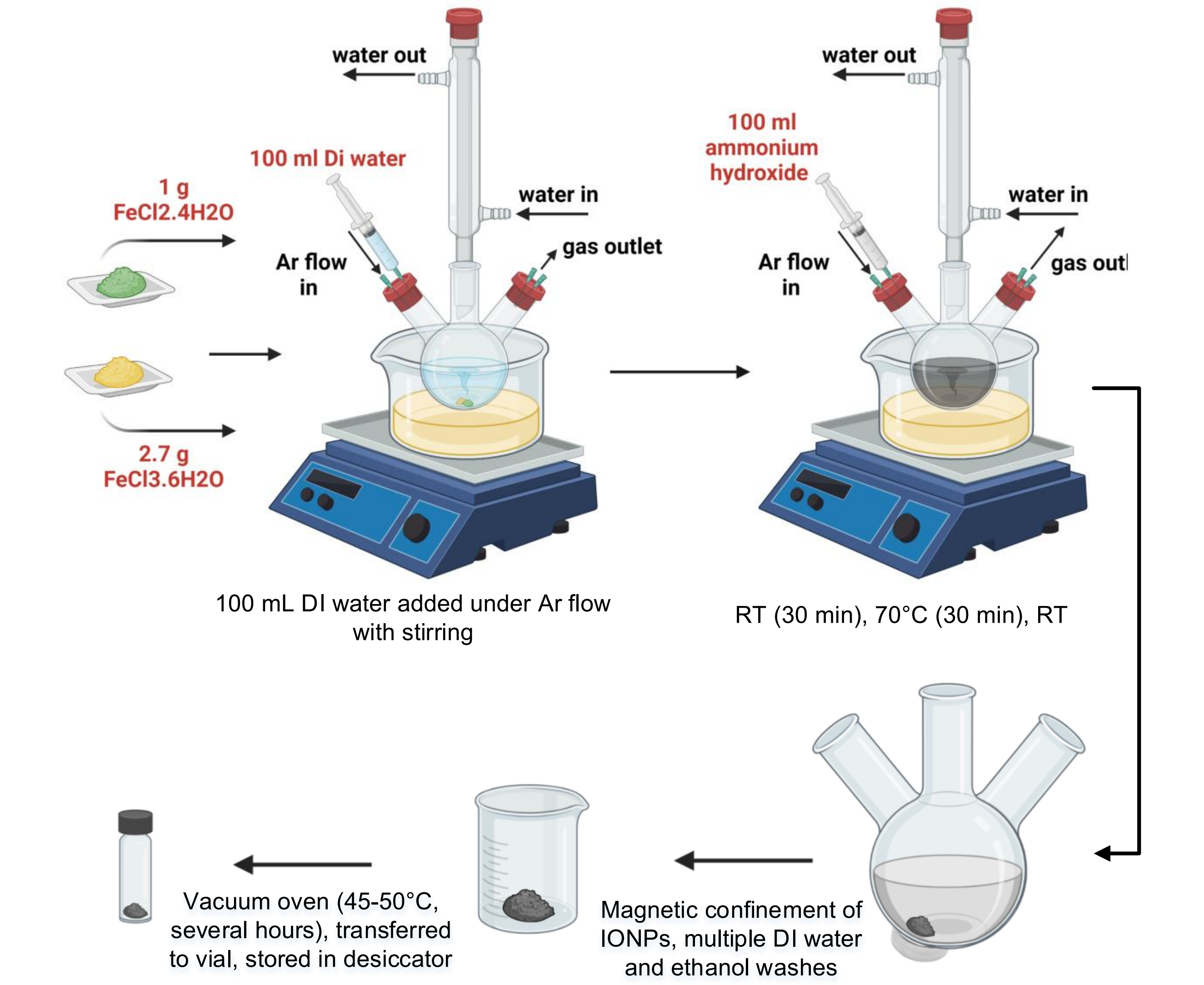

Figure S2. Schematic representation of the co-precipitation method used for synthesizing magnetic IONPs under an inert argon atmosphere. Iron (II) chloride tetrahydrate and iron (III) chloride hexahydrate were combined and reacted with ammonium hydroxide to produce dark-colored IONPs, which were subsequently purified with water and ethanol washes, dried, and stored for further use. Adapted from methods described in [28 and 29].

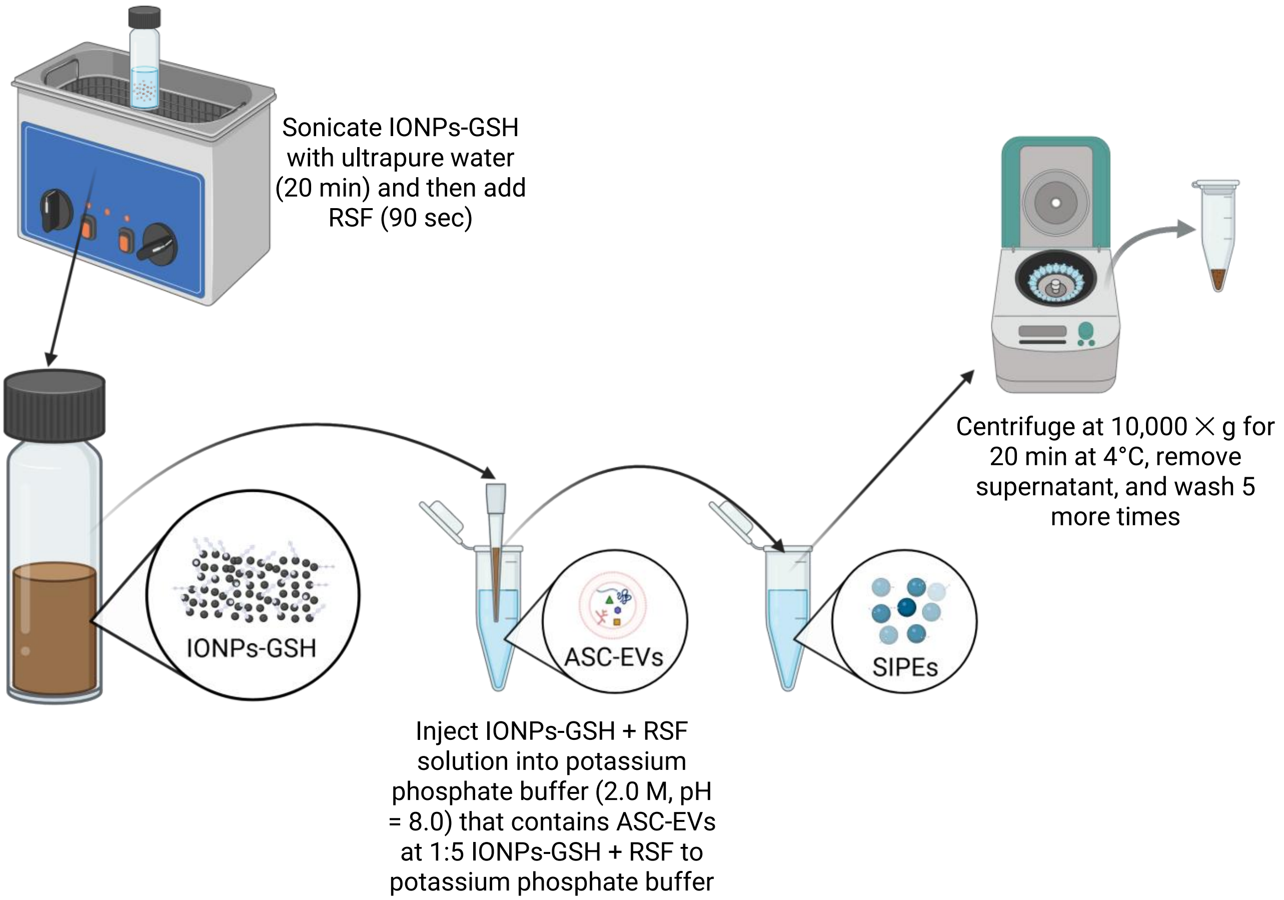

Figure S3. Schematic representation of fabrication of SIPEs. IONPs-GSH were mixed with ultrapure water and sonicated for 20 minutes. Then RSF was added and sonicated for 90 seconds. The IONP-GSH + RSF solution was injected 1:5 into potassium phosphate buffer that contains the ASC-EVs. This reaction precipitates the SIPEs. SIPEs are then centrifuged and washed a total of 6 times. Protocol modified from [28].

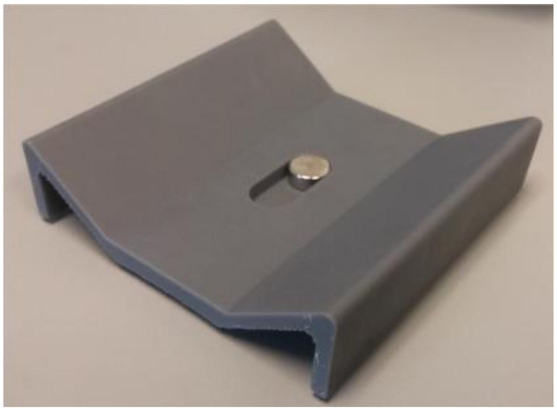

Figure S4. 3D printed bed for rat studies with removable diametric magnet in place.

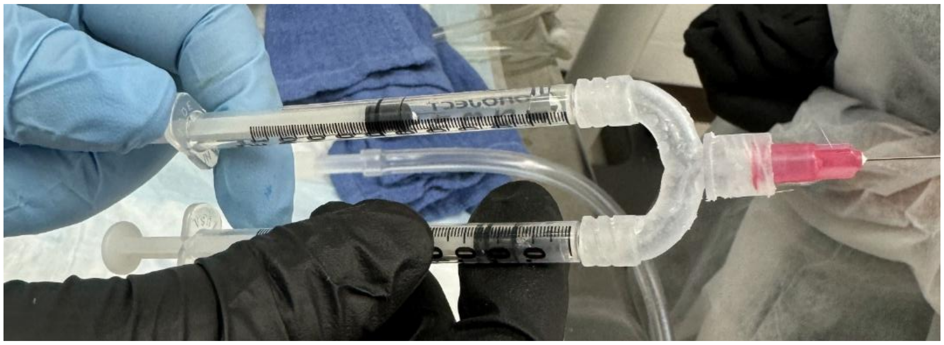

Figure S5. 3D printed bifurcation syringe mixer for simultaneously mixing fibrinogen (with SIPEs or SIMPs) and thrombin.

Injection of SIMPs

A custom 3D printed bed (**Figure S4**) and bifurcation (**Figure S5**) were made for in vivo injection of SIMPs.

Results

Nanoparticle Tracking Analysis

Before loading the EVs into SIPEs, NTA was performed to assess EV count and size of the RoosterBio Inc EVs. Total concentration (area under the curve) was calculated to be 8.57E9 ± 0.27E9 particles/mL with an average diameter of 144.9 nm ± 47.4 nm (**Figure** S6).

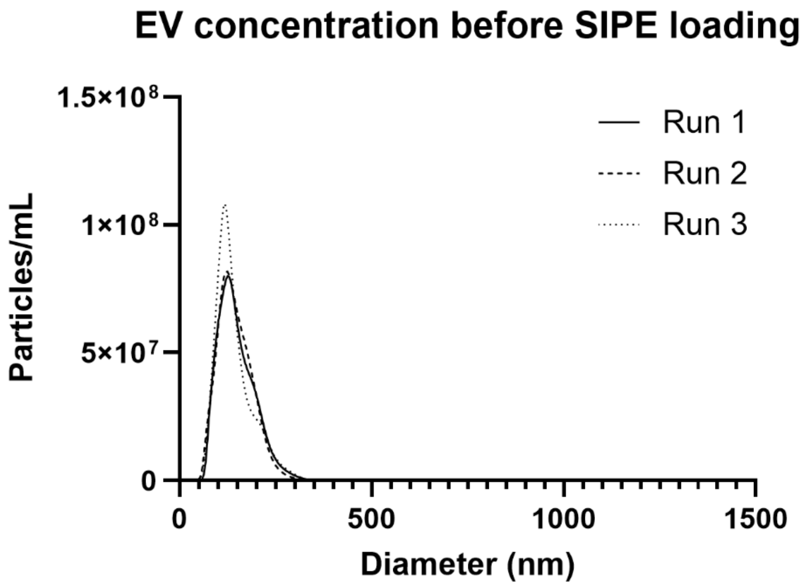

Figure S6**. NTA of EVs demonstrated relatively universal distribution of EVs across the 3 runs.** EVs had relatively the same peak across all 3 runs, indicating relatively consistent readings.

MicroBCA of EVs

ASC-EVs diluted with DPBS had relatively consistent protein concentration regardless of EV particle count (**Figure S7**) (n =3, 1E9 particles/mL: 8.09 ± 1.78 µg/mL, n = 2, 2E9 particles/mL: 10.15 ± 0.56 µg/mL, and n = 3, 3E9 particles/mL: 9.05 ± 0.50 µg/mL). However, ASC-EVs mixed and diluted with RIPA had increasing amounts of protein with increasing numbers of EVs (n = 3, 1E9 particles/mL: 6.87 ± 1.80 µg/mL, n = 3, 2E9 particles/mL: 16.55 ± 3.39 µg/mL, n = 2, 3E9 particles/mL: 26.00 ± 0.10 µg/mL). Additionally, comparing DPBS to the respective RIPA groups also showed significance once the particle count reached 2E9 particles/mL (1E9: $p = 0.0513$, 2E9: $p = 0.0010$, and 3E9: $p < 0.0001$).

Western Blot

ApoB was used as a negative control for EVs, as it should not be present in EVs; MSC lysate however should be positive for ApoB. As shown in **Figure S8,** RoosterBio EVs did not demonstrate presence of ApoB while the MSC lysate contained ApoB, indicating that this isolate was free from ApoB contamination.

ExoCheck

The RoosterBio EVs were positive for all EV markers, indicating that these particles were EVs (**Figure S9**). Details regarding what each marker checks for can be found in **Table S1**.

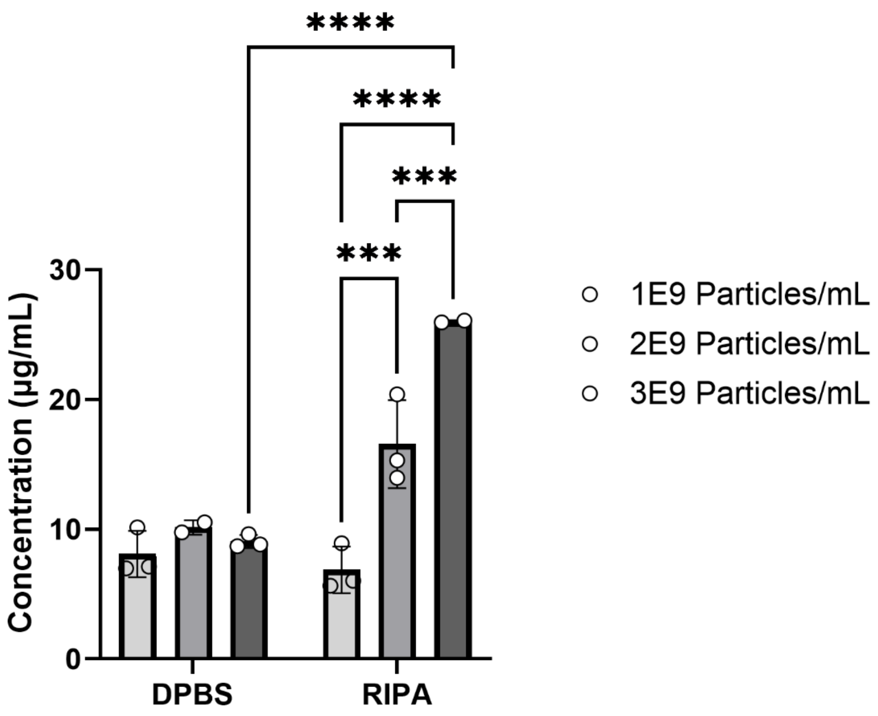

Figure S7. **EVs lysed with RIPA had increased encapsulated protein but non-encapsulated protein remained the same.**EVs were diluted with either DPBS or RIPA to see protein of EVs vs. encapsulated protein within EVs. Only once the concentration of EVs reached 2E9 particles/mL was a detectable difference seen between DPBS groups and RIPA groups.

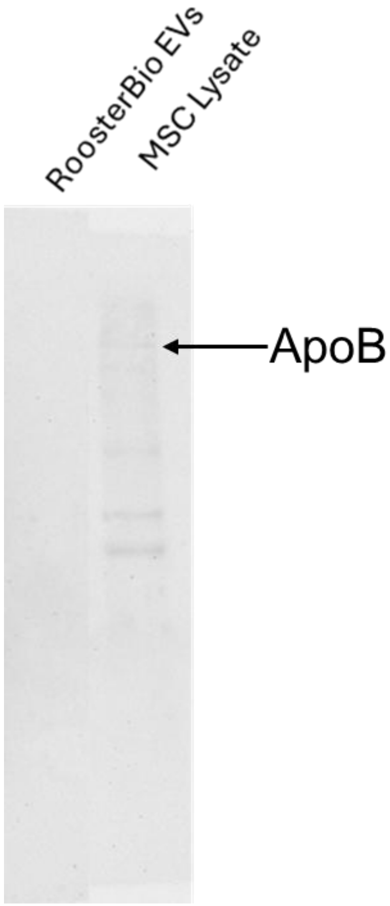

Figure S8. **Western blot of ApoB of RoosterBio EVs.** ApoB was used to stain for contaminating apolipoproteins, which should be absent in purer EV isolations. The RoosterBio EVs did not stain for ApoB while the MSC lysate contained ApoB, indicating that these EVs were pure.

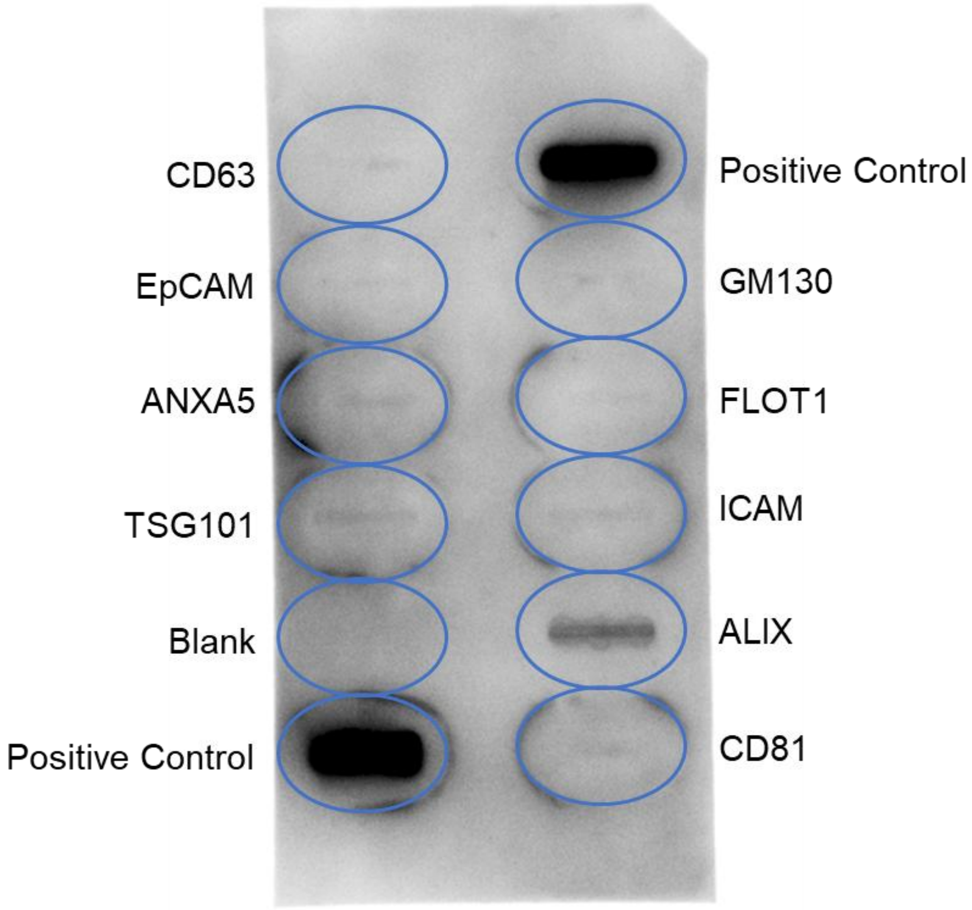

Figure S9. ExoCheck analysis of RoosterBio EVs. ExoCheck demonstrated a small presence of all EV markers, with a large presence of ALIX. In comparison to the blank (negative control), where there is no signal, there is a small positive signal from all EV markers indicated. CD63 and CD81, two tetraspanins, appeared to have similar expression. ALIX had the highest expression of all markers followed by TSG101. There are faint but comparable amounts of ICAM and ANXA5. GM130, EpCAM, and FLOT1 all have faint expression but less than other markers. As EVs can contain proteins, potential residual golgi associated markers could be present. Each marker is described in Table 3 along with the protein type each is in respect to the MISEV guidelines.

Table S1. Table of markers provided with ExoCheck kit (CD63, EpCam, ANXA5, TSG101, CM130, FLOT1, ICAM1, ALIX, and CD81) and their relation to each category from the MISEV guidelines. Descriptions were adapted from MISEV 2018 and 2023 guidelines.

Marker	Description
CD63	Non-tissue specific tetraspanin (1a)
EpCAM	Epithelial cell specific transmembrane or GPI-anchored protein (1b)
ANXA5	Cytosolic protein with lipid or membrane protein-binding ability, accessory protein (2a)
TSG101	Cytosolic protein with lipid or membrane protein-binding ability, ESCRT-I/II/III (2a)
GM130	Subtype of EVs, secretory pathway (ER or Golgi apparatus) (4c)
FLOT1	Cytosolic protein with lipid or membrane protein-binding ability, Flotillin-1 (2a)
ICAM1	Intercellular adhesion molecule 1
ALIX	Cytosolic protein with lipid or membrane protein-binding ability, accessory protein (2a)
CD81	Non-tissue specific tetraspanin (1a)

Characterization of EVs Provided by RoosterBio

RoosterBio provided rigorous characterization of their ASC-EVs including particle counts (**Figure S10** & **Table S2**), protein quantification (**Figure S11** & **Table S3**), EV purity (**Figure S12** & **Table S4**), and surface and cytosolic markers (**Figures S13 –S17** & **Tables S5 – S9**). EVs were at a concentration of 8.5 × 10^9 particles/mL with an average median size of 149.4 nm. They had a protein concentration of 81.7 µg/mL and an EV purity of 97.7%. ASC-EVs were positive for CD9, CD63, CD81, ALIX, and TSG101. Additionally, ASCs used to produce ASC-EVs had quantifiable concentrations of TIMP-1 and TIMP-2 in the cytokine secretions (**Figure S18**).

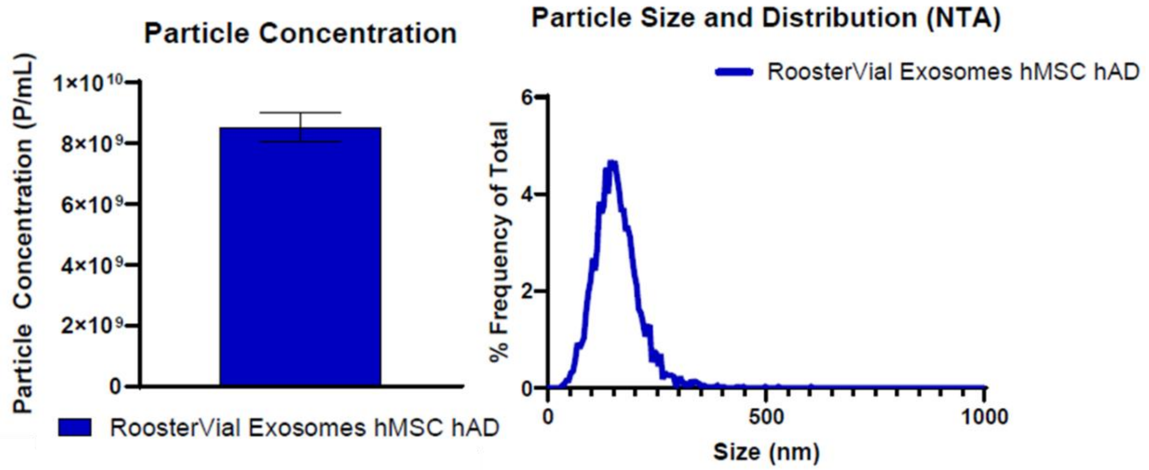

Figure S10. Particle concentration and size distribution of RoosterBio EVs provided by RoosterBio. Particles were concentrated at 8.5 × 10^9 particles/mL with an average diameter of 149.4 nm, as described in **Table S2**.

Table S2. Particle concentration and size distribution of RoosterBio EVs provided by RoosterBio over 3 different runs. Average particle count was 8.0 × 10^9 particles/mL and average diameter size of 149.4 nm.

Sample Name	Raw Particle Concentration (Particle/mL)	Dilution factor	Adjusted Particle Concentration (P/mL)	Average Particle Concentration (P/mL)	SD	%CV
RoosterVial Exosomes, hMSC, hAD	1.0 × 10^8	80	8.0 × 10^9	8.5 × 10^9	4.6 × 10^8	5.4
	1.1 × 10^8	80	8.8 × 10^9			
	1.1 × 10^8	80	8.8 × 10^9			
			Raw Median Size (nm)	Average Median Size (nm)	SD	%CV
			149.7, 154.4, 144.2	149.4	5.1	3.4

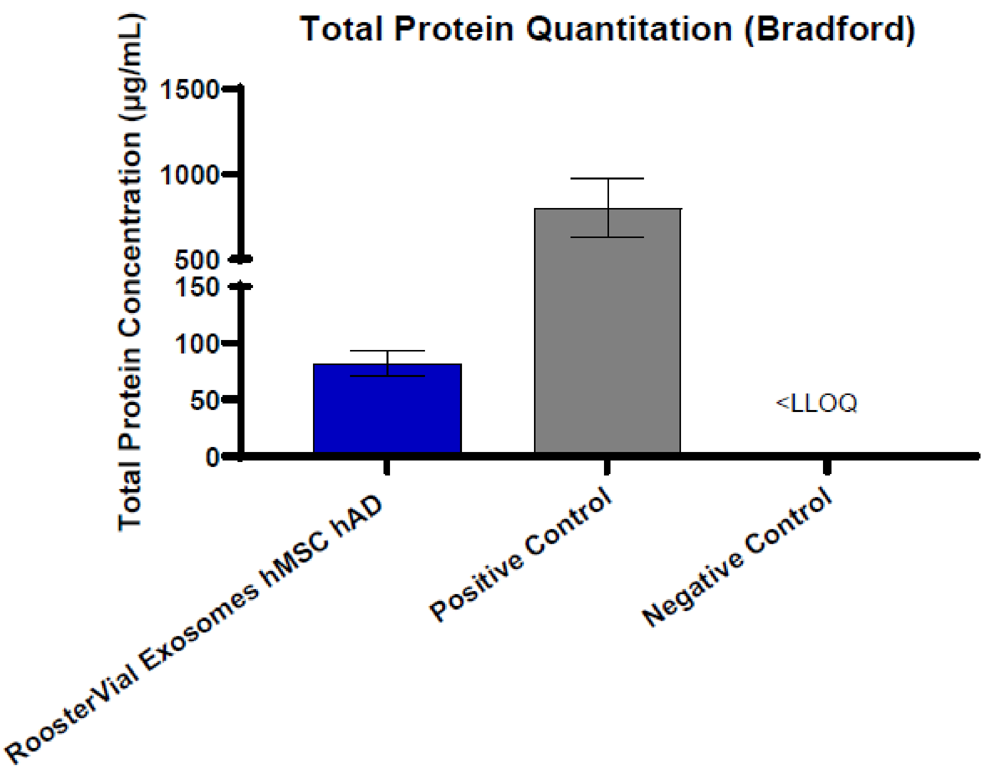

Figure S11. Total Protein Quantification in EVs provided by RoosterBio. Average protien concentration was 81.7 µg/mL across three replicates; the positive control was much higher while none was detected in the negative control. More specific details in **Table S3**.

Table S3. Table of protein concentration of EVs provided by RoosterBio. Average protein concentration was 81.7 µg/mL across three replicates; the positive control was much higher while none was detected in the negative control. LLOQ = lower limit of quantification

Sample Name	Adjusted Protein Concentration (µg/mL)	Average Protein Concentration (µg/mL)	SD	%CV
RoosterVial Exosomes, hMSC, hAD	87.7	81.7	11.4	14.0
	88.9			
	68.5			
Positive Control	1004.7	802.0	176.8	22.0
	722.0			
	679.4			
Negative Control	<LLOQ	<LLOQ	N/A	N/A
	<LLOQ			
	<LLOQ			

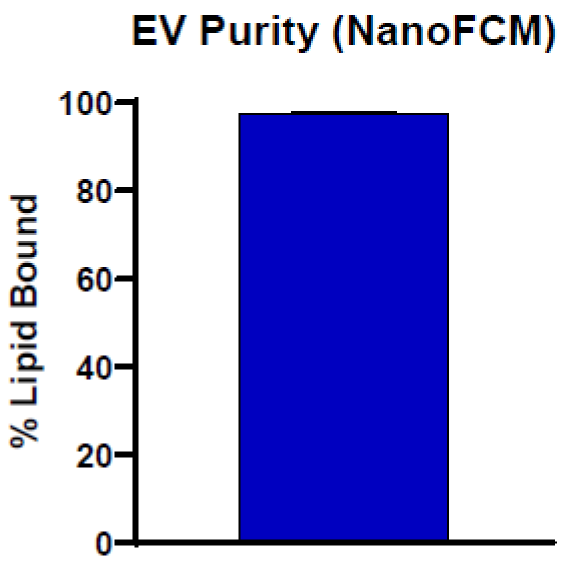

Figure S12. EV purity determined from RoosterBio. The number of lipid bound replicates was an average of 97.7%, indicating good purity of the isolation. More information can be found in **Table S4**.

Table S4. Number of lipid bound replicates to determine EV purity provided by RoosterBio. With an average lipid bound percentage of 97.7, these EVs were pure.

Sample Name	Average Number of Events	Lipid Bound Replicates (%)	Mean Lipid Bound (%)	SD	%CV
RoosterVial Exosomes, hMSC, hAD	4481	97.8	97.7	0.2	0.2
		97.5			
		97.8			

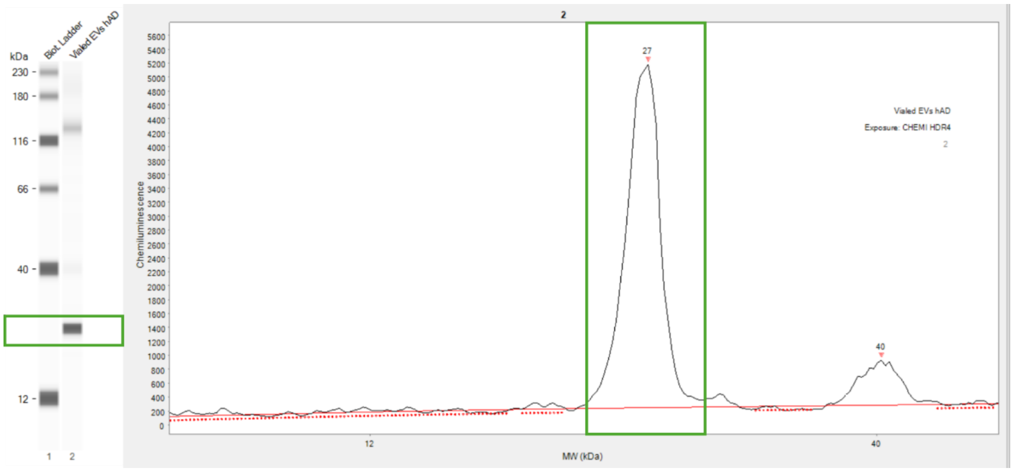

Figure S13. Surface marker expression of CD9, provided by RoosterBio. Using a Capillary Western Blot – Jess, CD9 was detected in the EVs. The green box on the left indicates the band in respect to the molecular weight, and the associated range expected for this particular marker. The green box on the right shows the actual molecular weight and the chemiluminescence. More details can be found in **Table S5**.

Table S5. Surface marker expression information of CD9 by RoosterBio. Using a Capillary Western Blot – Jess, CD9 was detected in the EVs.

Sample ID	Color Code	CD9 Molecular Weight Range (kDa)	Actual Molecular Weight (kDa)	Particles Loaded (P)	%CV	Protein Loaded /Well (µg)	Protein Loaded /Cap. (µg)	Chemiluminescent Signal
RoosterVial Exosomes, hMSC, hAD		22-35	27	7.86×10^5	0.1	0.1	0.002	4986.2

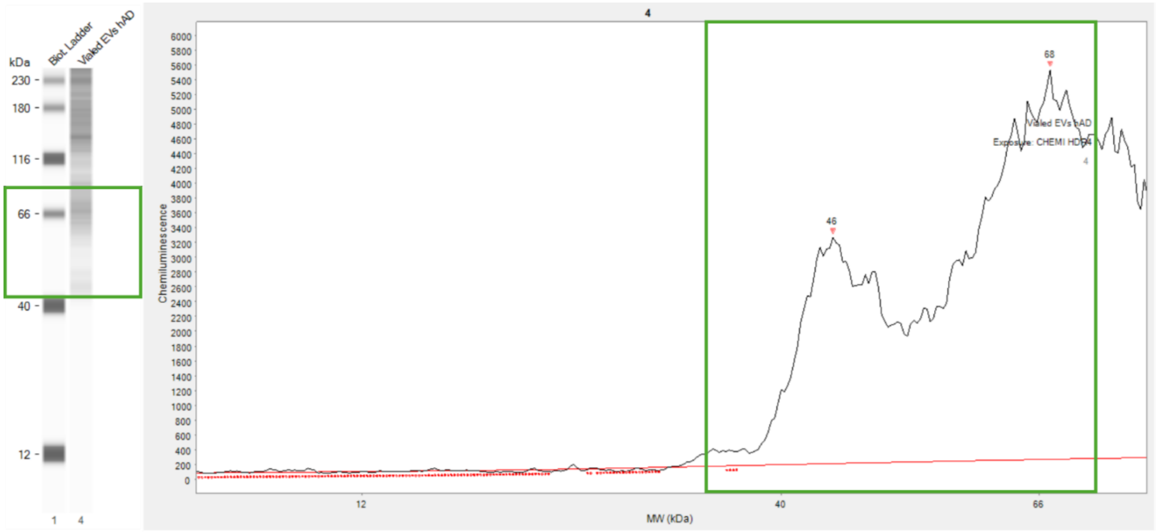

Figure S14. Surface marker expression of CD63, provided by RoosterBio. Using a Capillary Western Blot – Jess, CD63 was detected in the EVs. The green box on the left indicates the band in respect to the molecular weight, and the associated range expected for this particular marker. The green box on the right shows the actual molecular weight and the chemiluminescence. The two peaks are where CD63 can be expected molecular weight wise, as they are both within the expected range. More details can be found in **Table S6.**

Table S6. Surface marker expression information of CD63 by RoosterBio. Using a Capillary Western Blot – Jess, CD63 was detected in the EVs.

Sample ID	Color Code	CD63 Molecular Weight Range (kDa)	Actual Molecular Weight (kDa)	Particles Loaded (P)	%CV	Protein Loaded /Well (µg)	Protein Loaded /Cap. (µg)	Chemiluminescent Signal
RoosterVial Exosomes, hMSC, hAD		22-65	46-68	7.86×10^5	0.1	0.1	0.002	2532.2<br>4835.6

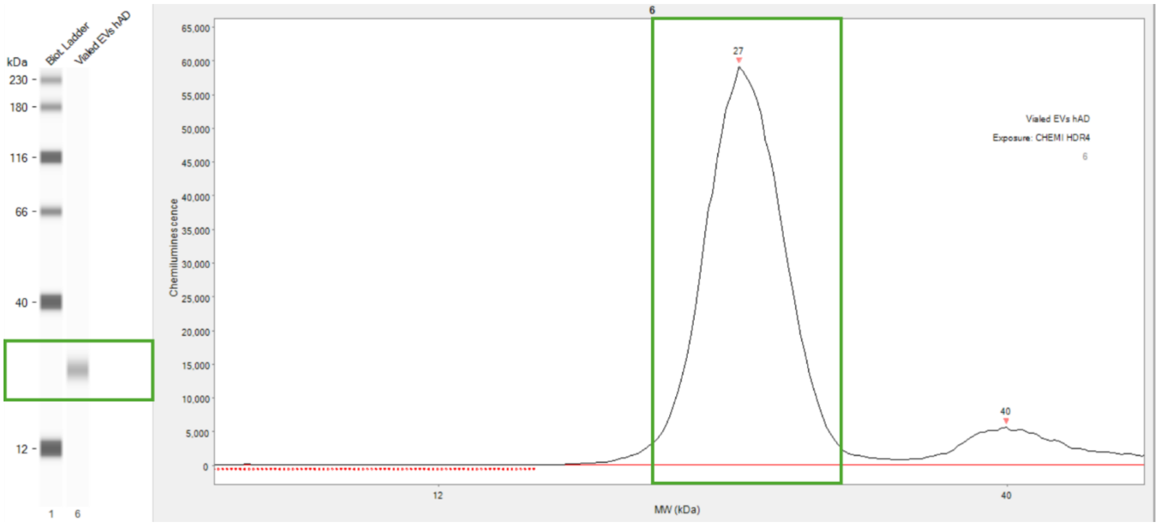

Figure S15. Surface marker expression of CD81, provided by RoosterBio. The green box on the left indicates the band in respect to the molecular weight, and the associated range expected for this particular marker. The green box on the right shows the actual molecular weight and the chemiluminescence. Using a Capillary Western Blot – Jess, CD81 was detected in the EVs. More details can be found in **Table S7.**

Table S7. Surface marker expression information of CD81, provided by RoosterBio. Using a Capillary Western Blot – Jess, CD81 was detected in the EVs.

Sample ID	Color Code	CD81 Molecular Weight Range (kDa)	Actual Molecular Weight (kDa)	Particles Loaded (P)	%CV	Protein Loaded /Well (µg)	Protein Loaded /Cap. (µg)	Chemiluminescent Signal
RoosterVial Exosomes, hMSC, hAD		22-35	27	7.86×10^5	0.1	0.1	0.002	58180.4

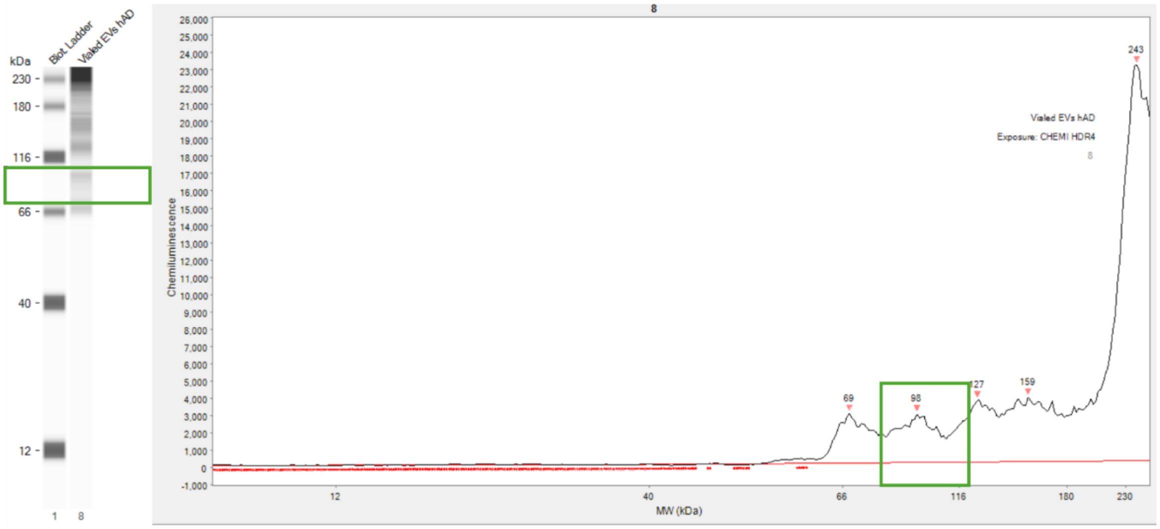

Figure S16**. Cytosolic protein expression of ALIX, provided by RoosterBio.** Using a Capillary Western Blot – Jess, ALIX was detected in the EVs. The green box on the left indicates the band in respect to the molecular weight, and the associated range expected for this particular marker. The green box on the right shows the actual molecular weight and the chemiluminescence. Only peak is chosen as it is within the range expected. More details can be found in **Table S8.**

Table S8. **Surface marker expression information of ALIX, provided by RoosterBio.** Using a Capillary Western Blot – Jess, ALIX was detected in the EVs.

Sample ID	Color Code	ALIX Molecular Weight Range (kDa)	Actual Molecular Weight (kDa)	Particles Loaded (P)	%CV	Protein Loaded /Well (µg)	Protein Loaded /Cap. (µg)	Chemiluminescent Signal
RoosterVial Exosomes, hMSC, hAD		65-100	98	7.86×10^5	0.1	0.1	0.002	2476.4

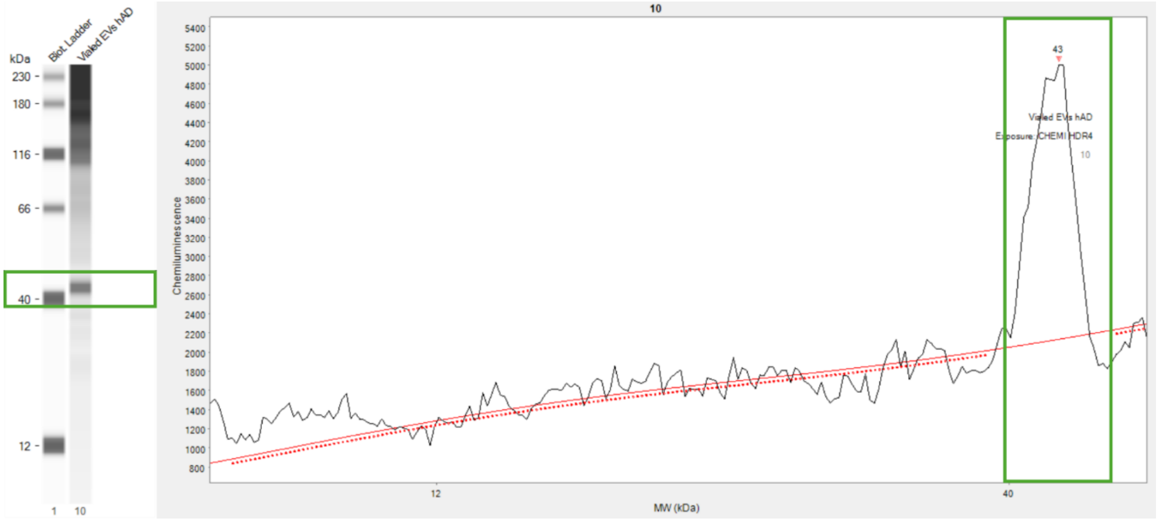

Figure S17. Cytosolic protein expression of TSG101, provided by RoosterBio. The green box on the left indicates the band in respect to the molecular weight, and the associated range expected for this particular marker. The green box on the right shows the actual molecular weight and the chemiluminescence. Using a Capillary Western Blot – Jess, TSG101 was detected in the EVs. More details can be found in **Table S9.**

Table S9. **Cytosolic protein expression information of TSG101, provided by RoosterBio.** Using a Capillary Western Blot – Jess, TSG101 was detected in the EVs.

Sample ID	Color Code	TSG101 Molecular Weight Range (kDa)	Actual Molecular Weight (kDa)	Particles Loaded (P)	%CV	Protein Loaded /Well (µg)	Protein Loaded /Cap. (µg)	Chemiluminescent Signal
RoosterVial Exosomes, hMSC, hAD		44-46	43	7.86×10^5	0.1	0.1	0.002	2531.1

Characterization of Cytokines Secreted from Parent ASCs of RoosterBio ASC-EVs

Angiogenic cytokines secreted by ASCs were characterized by RoosterBio (**Figure S18**). Both TIMP-1 and TIMP-2 were detected in this analysis.

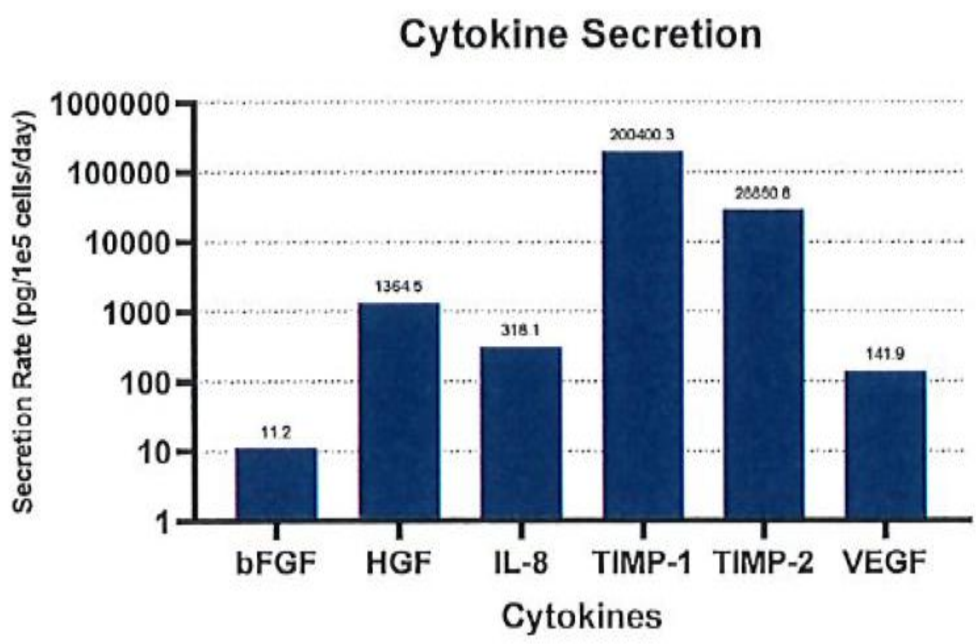

Figure S18. Angiogenic cytokines secreted from ASCs were analyzed using a MultiPlex ELISA. Two cytokines of interest are TIMP-1 and TIMP-2, as they play a large role in modulating degradation. There was detection of bFGF, HGF, IL-8, and VEGF as well.

Uptake of Encapsulated ASC-EVs from RoosterBio

Following 24 hours of incubation with SMCs in fibrin gels, SIPEs were able to release fluorescently labelled ASC-EVs purchased from RoosterBio. These ASC-EVs were then taken up by SMCs. ASC-EVs had more fluorescent signal in the Cy5 channel compared to dye controls (**Figure S19**).

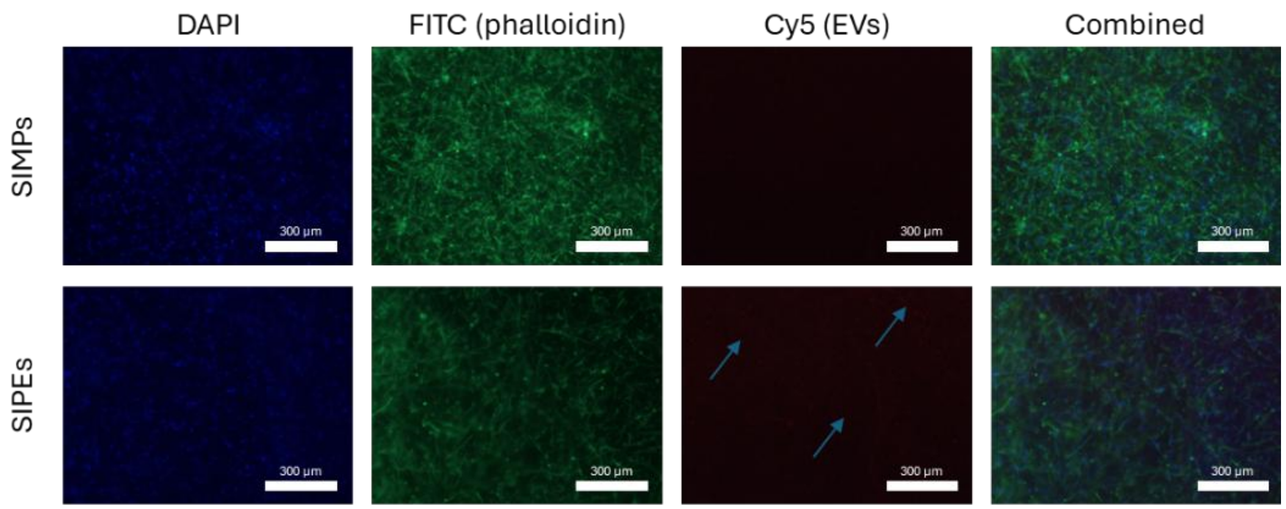

Figure S19. EVs can be released and taken up by SMCs in 3D fibrin gel constructs. SIPEs and SIMPs were loaded with EVs or a dye control respectively and then cultured for 24 hours with SMCs. Cy5 signal appears more vibrant in the SIPE group compared to SIMPs.

Migration and Proliferation Assays including Controls

Migration assays (**Figure S20A**) and proliferation assays (**Figure S20B**) showed increased migration and proliferation with ASC-EVs compared to DPBS controls. Supplemented media (positive control) showed increased migration compared to the unsupplemented media (negative control). Fully supplemented media also caused the highest amount of proliferation.

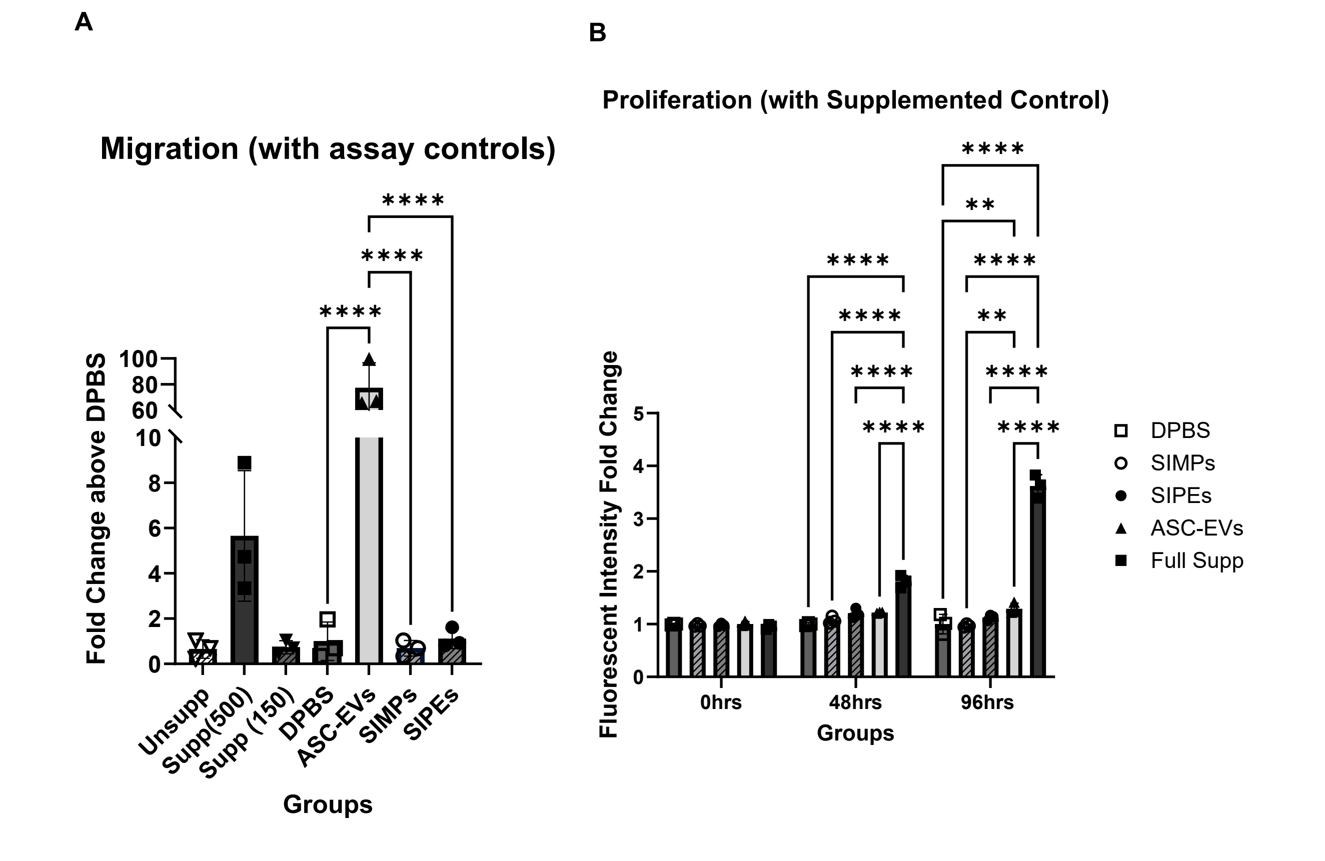

Figure S20. ASC-EVs increased migration (A) and proliferation (B) of SMCs. These graphs also show that the assay worked, according to the positive and negative controls.
